# Parasite Specific Antibody Increase Induced by an Episode of Acute *P*. *falciparum* Uncomplicated Malaria

**DOI:** 10.1371/journal.pone.0124297

**Published:** 2015-04-23

**Authors:** Mark Kaddumukasa, Catherine Lwanira, Allan Lugaajju, Elly Katabira, Kristina E. M. Persson, Mats Wahlgren, Fred Kironde

**Affiliations:** 1 Department of Medicine, College of Health Sciences, Makerere University, Kampala, Uganda; 2 College of Health Sciences, Makerere University, Kampala, Uganda; 3 Department of Microbiology, Tumour and Cell Biology, Karolinska Institutet, Stockholm, Sweden; 4 Department of Laboratory Medicine, Lund University, Stockholm, Sweden; 5 Habib Medical School, IUIU, Kampala, Uganda; Universidade Federal de Minas Gerais, BRAZIL

## Abstract

**Introduction:**

There is no approved vaccine for malaria, and precisely how human antibody responses to *malaria parasite * components and potential vaccine molecules are developed and maintained remains poorly defined. In this study, antibody anamnestic or memory response elicited by a single episode of *P*. *falciparum* infection was investigated.

**Methods:**

This study involved 362 malaria patients aged between 6 months to 60 years, of whom 19% were early-diagnosed people living with HIV/AIDS (PLWHA). On the day malaria was diagnosed and 42 days later, blood specimens were collected. Parasite density, CD4^+ ^ cells, and antibodies specific to synthetic peptides representing antigenic regions of the *P*. *falciparum* proteins GLURP, MSP3 and HRPII were measured.

**Results:**

On the day of malaria diagnosis, Immunoglobulin (IgG) antibodies against GLURP, MSP3 and HRP II peptides were present in the blood of 75%, 41% and 60% of patients, respectively. 42 days later, the majority of patients had boosted their serum IgG antibody more than 1.2 fold. The increase in level of IgG antibody against the peptides was not affected by parasite density at diagnosis. The median CD4^+^ cell counts of PLWHAs and HIV negative individuals were not statistically different, and median post-infection increases in anti-peptide IgG were similar in both groups of patients.

**Conclusion:**

In the majority (70%) of individuals, an infection of *P*. *falciparum* elicits at least 20% increase in level of anti-parasite IgG. This boost in anti-*P*. *falciparum* IgG is not affected by parasite density on the day of malaria diagnosis, or by HIV status.

## Introduction

*Plasmodium falciparum* malaria is a leading cause of morbidity and mortality among children in sub-Saharan Africa [1]. Humoral immune responses are believed to be critical to effective immunity against *Plasmodium falciparum* [2] and play a key role in the development of naturally acquired immunity to malaria [3, 4]. However, protective immunity to malaria takes years to develop and requires repeated infections [5]. Earlier studies showed that transfer of serum or antibody preparations from partially immune residents of endemic regions to non-immune individuals has beneficial anti-malarial activity [6, 7] and that malaria specific antibodies are associated with protection against malaria [8, 9]. In general, high antibody levels are associated with reduced susceptibility to clinical malaria [10, 11]. However, how the malarial antibodies are acquired, maintained, and boosted by regular parasite infections in endemic areas is little understood. Yet, if natural boosting of anti-malaria immunity is well elucidated, the information can be of great value in developing robust vaccines against malaria. Earlier studies have shown that, when individuals are exposed to a new malaria infection, parasite-specific antibody levels usually increase markedly within 1 or 2 weeks after the onset of symptoms [12, 13]. The boosted antibodies normally decline fast after an infection is resolved, suggesting that protective memory for specific antibody response is not efficiently formed or is functionally impaired [14]. However, moderate antibody levels persist in many individuals. Such persistence has been shown to increase with age among young children [15]. Nevertheless, little is known about how ordinary *P*. *falciparum* infections work to build antibody responses to candidate vaccine molecules. We know very little about the extent to which natural infections will boost antibodies generated by candidate malaria vaccines. Candidate blood-stage vaccines include GMZ2 which has been tested in children and adults [16, 17] and comprises glutamine rich protein (GLURP) and merozoite surface protein 3 (MSP3) [9, 14, 18]. MSP3 and GLURP are believed to be involved in erythrocyte invasion [19, 20] and are important vaccine candidates [21, 22]. Indeed, high levels of specific cytophilic antibodies against MSP3 and GLURP have been reported to be associated with protection against clinical malaria [23, 24].

A few clinical trials where whole sporozoite or trophozoite forms were used as vaccine showed that development of host protective immunity against malaria can be achieved although such vaccination would require large numbers of parasites [25, 26].

To gain further insight into the boosting of parasite specific antibody response following acute *P*. *falciparum* uncomplicated malaria, we investigated the antibody anamnestic response against three synthetic peptides [22] representing MSP3, GLURP, and Histidine Rich Peptide—II (HRPII) as well as parasitized erythrocyte (PE) lysate antigens. For this, we identified blood-smear positive *P*. *falciparum* infected patients on day 0 (baseline), and collected venous blood for determination of parasite density, CD4^+^ cell counts and sero-prevalence of antibodies aganist the synthetic peptides and to antigens in PE lysate. The patients were treated with artemether—lumefantrine (co-artem) according to standard treatment guidelines. At day 42, blood was collected from the convalescent patients to determine post-infection levels of specific Immunoglobulin (IgG) and sero-prevalence to the three peptides. The relationship between the levels of anti-peptide antibodies for day 0 and day 42 was assumed to represent a reasonable measure of secondary anti-*P*. *falciparum* antibody boost or increase after an acute episode of uncomplicated malaria.

## Materials and Methods

### Study area and subject enrolment

The study was conducted at Kasangati Health Centre (KHC) which is about 20 km, north east of Kampala the capital city of Uganda. Patients were recruited from the peri-urban villages located within 10 km of KHC. Kasangati lies within a moderate transmission area, with a peak transmission after the two main rainy seasons (February-March and September-October) every year. This study was conducted from November 2010 to January 2011 after the September—October rains. Consecutive sampling was used to enrol a prospective cohort of participants. Eligibility criteria included: uncomplicated malaria as defined previously [27]. In brief, inclusion criteria were history of fever in the previous 24 hr, axillary temperature ≥ 37.5°C, haemoglobin > 5 g/dl, *P*. *falciparum* mono-infection at any parasitemia, and informed written consent from participants or their parents or guardians for minors, agreement to come to the study clinic for 42 day follow up, age: more than 6 months. A detailed clinical examination was performed and data entered into a pre-tested study questionnaire. The first dose of Artemether-Lumefantrine was administered under direct observation to ensure compliance. If a patient vomited within 30 min, the dose was repeated. The remaining doses were administered at home unsupervised.

#### Human subject protection and ethics approval statement

A written informed consent was sought from all study participants before they were enrolled into the study. The study was approved by the Makerere University, School of Medicine, Research and Ethics Committee (SOMREC) and the Uganda National Council of Science and Technology (UNCST), 2007–045.

### Clinical investigations and follow-up

On the day of recruitment, a finger prick blood specimen was taken and thick smears prepared and treated with Giemsa stain. Participants found to be smear positive for *P*. *falciparum* mono-infection were subsequently enrolled and a venous blood sample taken for sera and estimation of CD4^+^ cells. Follow-up of patients was arranged for day 42 and participants were asked to return to the clinic whenever they felt unwell within the subsequent 6 weeks. Follow-up consisted of history taking, a physical examination and blood smear examination.

During the day 42 visit, a blood specimen was collected to re-assess the antibody levels. Standardized treatment was provided according to the national treatment guidelines. Patients who failed to return on day 42 were visited at their homes by our social worker and brought to the health unit for examination and medical care. Day 42 was selected as suitable for assessing the secondary antibody response after *P*. *falciparum* infection [28] and determining clinical outcome according to the WHO treatment guidelines [29, 30].

### CD4 ^+^ T cell counts and examination of stained blood smears for parasites

Thick smears were stained with 10% Giemsa stain for 10 min, sexual and asexual parasites were counted against every 200 white blood cells (WBCs). Results were multiplied by 40 to determine the parasite density (parasites/μL), assuming a normal WBC count of 8000 per μl [31, 32]. A smear result was judged to be negative if no parasites were seen after review of 100 high-power (400-fold magnification) fields. Final microscopy results were based on a rigorous quality control system, with re-reading of all blood smears by a second microscopist and resolution of any discrepancies by a third microscopist. The CD4^+^ T-cell counts were measured by a FACS Counter (Becton Dickinson, San Jose, CA, USA).

### Measurement of serum IgG antibodies

*P*. *falciparum* serum antibodies to peptides MSP3, HRP II and GLURP were measured by enzyme linked immunorsobent assay (ELISA) essentially as described before [23] using respective synthetic peptides as antigen and peroxidase-conjugated anti-human IgG or anti-human IgM as secondary antibody. Briefly, microtiter plate wells were coated with 0.5 to 1 microgram of peptide per well, incubated overnight at 4°C, and blocked with 5% skimmed milk for 2 hours at room temperature. Serum specimens were diluted 1:200, added in duplicates and incubated at room temperature for 1 h. Plates were washed 4 times between the coating and blocking steps. The microtiter plate wells were then incubated (45 min) with peroxidase-conjugated goat anti-human IgG (secondary antibody). Bound secondary antibody was quantified by developing colour with TMB (*3*, *3’*, *5*, *5’-Tetramethylbenzidine*) substrate. Optical density (OD) was read at 450 nm with a reference at 620 nm using a plate reader. Sera of known unexposed individuals were used as controls. All the ELISAs for a given antigen were performed on the same day and on the same ELISA plate for day 0 and day 42 antibody tests of the same individual to eliminate the day to day variations.

All samples with optical density readings above 1.4 were diluted further up to 1:1000 to ensure that ODs were less than 1.4. The OD obtained multiplied by the dilution factor. All specimens were analyzed twice or thrice in duplicates and the means of the ELISA OD used in the analysis. If the ELISA OD of an individual’s day 42 serum was higher than the same person’s corresponding value for the day 0 specimen, the increase was regarded as reflecting a positive antibody anamnestic response or boost. The ratio (D42 ELISA OD)/ (D0 ELISA OD) as well as % increase {(100 x (Day42 ELISA OD—Day0 ELISA OD)/Day0 ELISA OD} was then calculated.

### Synthetic peptides

Sero-reactivity to peptides of *P*. *falciparum* antigens was determined by ELISA using synthetic peptides which were coated onto ELISA microtiter plate wells. Amino acid sequences of the synthetic peptides [14, 22, 33, 34] representing *P*. *falciparum asexual* blood stage proteins were; GLURP: (NH2) CGDKNEKGQHEIVEVEEILPEGC (CONH2), HRP II: (NH2)GCAHHAADAHHAADAHHAADAHHAADGC(CONH2), and MSP3: (NH2) AKEASSYDYILGWEFGGGVPEHKKEEN (CONH2).

### Statistical analysis and data presentation

Data were analysed using STATA version 11.0 (Stata Corporation, College Station, Texas, USA) and Graph pad Prism 5. Data were tested for normality using Shapiro-Wilk W test. Differences between medians of IgG levels on day 0 and day 42 were evaluated by Mann-Whitney test while those among three or more groups were assessed by the non-parametric Kruskal-Wallis test. Correlations were identified by Spearman rank test using actual or logarithmically transformed values P values <0.05 were regarded as statistically significant.

## Results

In this study, peripheral blood and serum of *P*. *falciparum* infected individuals suffering from acute uncomplicated malaria were obtained to determine i) level of CD4^+^ lymphocytes, ii) IgG sero-reactivity to antigens of lysed parasitized erythrocytes and synthetic polypeptides representing merozoite proteins GLURP, HRP II and MSP3 all of which are important parasite antigens or vaccine candidate molecules [16, 22]. Anti-malarial treatment was then started and 42 days later, the convalescent patients donated another blood specimen which was used to re-estimate the serum IgG against parasite antigens and synthetic peptides. The fold increase in level of anti-*P*. *falciparum* antibody was then determined by comparing levels of peptide-specific IgG for day 42 and day 0 as estimated by ELISA. The effect of CD4^+^ T cell levels and parasite density on magnitude of the sero-reactivity to synthetic peptides was analyzed.

### General Characteristics

In this study, 362 participants diagnosed with uncomplicated malaria were enrolled. About 58% (211/362) of the enrolled study participants were female (Table 1). Forty two percent (109/362) had not yet attended school for those aged less than five years or had no prior education training, 43% had attained primary level training while 15% had attained secondary level education. Among the study participants enrolled, nearly nineteen percent (68/362) were found to be HIV sero-positive. Of these HIV positive individuals, only seven percent (5/68) reported taking Cotrimoxazole prophylaxis therapy and none was on any anti retroviral therapy (ARV). 136 participants (38%) presented with symptoms of cough on day 0. Approximately 16% (57/362) of the study participants reported having taken an anti-helminthic treatment in the past one month before enrolling into this study. Only three percent (12/362) of the study participants reported that they had taken antimalarial treatment before coming to the health centre. Among the study participants, 67% (241/362) owned a mosquito net in their household and 61% (223/362) of the enrolled subjects slept under a mosquito net in the night preceding the day of enrolment (day 0). Only 38% (138/362) reported owning an insecticide treated bed net (ITN) while 8% (27/362) were not sure on whether their mosquito nets were treated. Enlargement of the spleen was observed in 17% (60/362) of the subjects. The median age of enrolled patients was 9.5 years (IQR: 3–21) and the reported duration of the malaria episode was 3 days (inter quartile range: 2–4). The median axillary temperature measured on day 0 was 37.95°C (IQR: 37.5–38°C).

**Table 1:** Characteristics of the study population (N = 362).

Characteristic	Number (%)
Sex	
Female	211 (57.8)
Age group	
Less than 5 years	124 (34.3)
6–15 years	121 (33.4)
>16 years	117 (32.3)
Level of education	
No education	109 (41.9)
Primary	111 (42.7)
Secondary	40 (15.4)
Cough on day 0	
Yes	136 (37.6)
No	226 (62.4)
Septrin prophylaxis^1^	
Yes	5 (16.6)
No	255(98.1)
Splenomegaly	
Palpable	60 (16.6)
Not palpable	302 (83.4)

^1^ denotes N = 260.

### Relationship between demographic characteristics and boosting after acute uncomplicated *P*. *falciparum* malaria

We evaluated the association of all baseline factors and post-infection increase in parasite-specific IgG antibody among the study participants (Table 2). Sex of the study participants and co-infection with HIV were not significantly associated with increase of antibody IgG against GLURP, HRPII and MSP3. Similarly, the duration of fever in days, use of antimalarial therapy before enrolment, self reported use of de-worming drugs, owning a mosquito net within the household and sleeping under a mosquito net in the night preceding the interview were not associated with post-infection increase in specific antibody IgG. However, the presence of a palpable spleen among the study participants was slightly associated with post-infection increase of IgG antibodies to GLURP and MSP3 (p = 0.030 and 0.031, respectively) while anti-HRPII antibodies were hardly affected by presence of palpable spleen.

**Table 2:** Multivariate analysis of subject characteristics and antibody boosting.

Subject characteristic	Anti-GLURP IgG		Anti-HRPII		Anti-MSP3 IgG	
	Estimate^1^	P^2^	Estimate^1^	P^2^	Estimate^1^	P^2^
Age group						
Less than 5 years	0	0	0	0	0	0
6–10 years	-0.17 (-0.46,0.12)	0.255	0.40 (-0.49,1.29)	0.367	0.28 (-0.12,0.68)	0.169
11–15 years	0.14 (0.50, 0.21)	0.437	0.57 (0.36, 1.49)	0.224	0.61 (0.13, 1.08)	0.013
>15 years	0.15 (-0.13, 0.43)	0.281	0.36 (-0.44, 1.15)	0.370	0.70 (0.31, 1.09)	0.001
Sex	-0.06 (-0.29, 0.16)	0.565	-0.22 (-0.81, 0.37)	0.460	0.12 (-0.20, 0.43)	0.467
HIV infection	-0.47 (-0.34, 0.24)	0.75	0.21 (-0.47, 0.90)	0.541	-0.30 (-0.69, 0.09)	0.137
Septrin prophylaxis	-0.28 (-0.58, 0.52)	0.92	0.77 (-1.56, 3.10)	0.510	-0.12 (-1.21, 0.96)	0.821
Duration of fever	0.04 (-0.02, 0.09)	0.163	-0.06 (-0.72, 0.47)	0.475	-0.07 (-0.15, 0.00)	0.064
Use of anti-malarial treatment before	-0.05 (-1.12, 0.17)	0.151	-0.69 (-2.43, 1.05)	0.431	-0.63 (-1.55, 0.29)	0.183
Use of deworming treatment	0.14 (-0.14, 0.43)	0.325	-0.07 (-0.86, 0.74)	0.862	-0.06 (-0.46, 0.33)	0.765
Owning a mosquito net in household	-0.22 (-0.46, 0.01)	0.06	0.22 (-0.37, 0.81)	0.465	-0.63 (-0.38, 0.25)	0.695
Sleeping under mosquito net in household	-0.22 (-0.25, 0.21)	0.874	0.27 (-0.32, 0.86)	0.367	0.08 (-0.23, 0.40)	0.580
Temperature	0.00 (-0.22, 0.22)	0.981	0.07 (-0.53, 0.66)	0.823	-0.12 (-0.43, 0.19)	0.435
Spleen	0.31 (0.03, 0.59)	0.030	0.84 (-0.05, 1.73)	0.063	0.42 (0.03, 0.80)	0.031
Malarial asexual parasitemia	0.07 (-0.01, 0.15)	0.108	-0.01 (-0.23, 0.21)	0.936	0.12 (0.08, 024)	0.036

^1^ denotes the coefficient/estimates.

^2^ denotes the p–value

### Sero-prevalence to synthetic peptides on day 0 and age dependence of anti-peptide IgG on days 0 and 42

**Fig 1:**
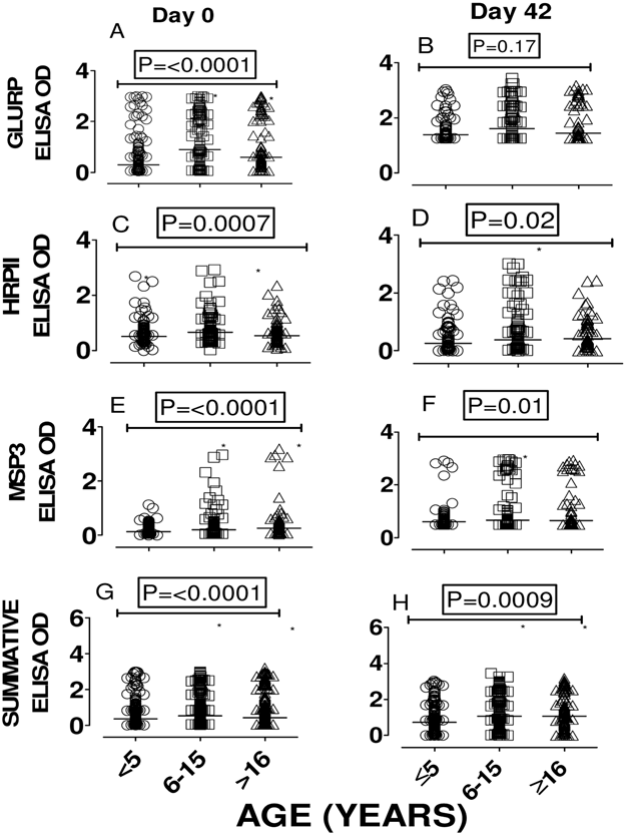
Age dependent post-infection antibody response to *P*. *falciparum* synthetic peptides. To determine increase in parasite-specific IgG after *P*. *falciparum* malaria attack, sera collected at diagnosis (day 0) and 42 days later (day 42) were analyzed by ELISA. Graphs A, C, E and G represent antibody levels on day 0 while graphs B, D, F and H represent antibody levels on day 42. The level of serum anti-peptide IgG on day 0 was age dependent, participants older than 16 years showing the highest level of serum anti-peptide IgG. The Kruskal-Wallis test results: GLURP (H = 30.7, 2 df, P = <0.0001); HRPII (H = 14.7, 2 df, P = <0.0007); MSP3 (H = 30.7, 2 df, P = <0.0001); cumulative (H = 39.9, 2 d.f, P = <0.0001. Likewise, on day 42, older participants (> 16 years) had higher levels of anti-peptide IgG. ‘Summative’ represents sum total of ELISA absorbances against all three peptides plotted against age.

On day 0, the frequencies of IgG-sero-reactivity against GLURP, MSP3 and HRP II peptides were 75% (271/360), 60% (217/360) and 41% (148/358), respectively. On the other hand, the corresponding levels for IgM sero-prevalence to GLURP, HRP II and MSP3 were 80% (207/259), 42% (108/257) and 22% (57/256), respectively. For day 0 blood specimens, anti-peptide IgG levels were significantly associated with age for all the three peptides “Figs 1A, 1C, 1E and 1G”, the highest age group (16 years and above) showing the highest IgG levels. Likewise, for day 42, the median IgG ELISA units were higher in the eldest age group (> 16 years) studied. “Figs 1B, 1D, 1F and 1H”

### Increase in serum anti-parasite IgG after a *P*. *falciparum* malaria attack

**Fig 2:**
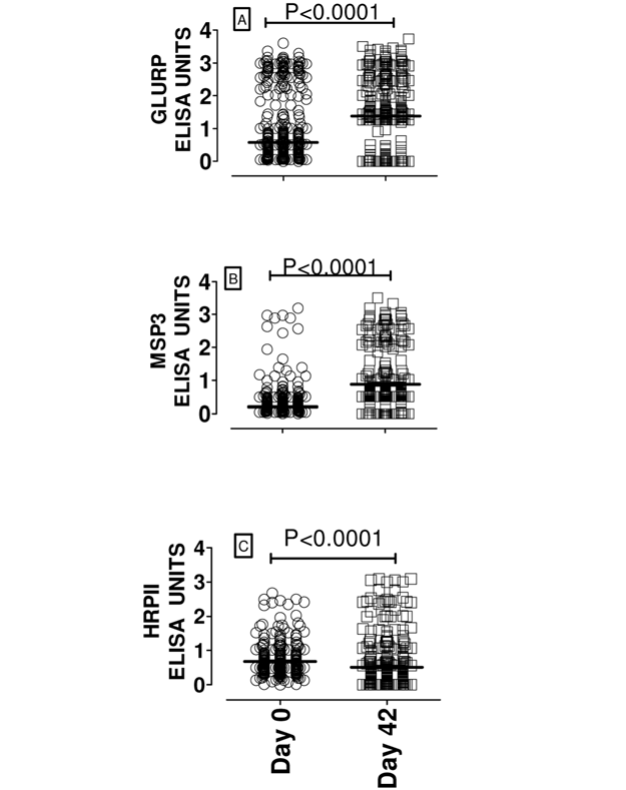
Post-infection increase in parasite-specific IgG. To evaluate memory-response Ab increase elicited by attack of *P*. *falciparum* malaria, levels of anti-parasite IgG for day 0 and day 42 were compared. A–C show the antibody IgG levels (ELISA units) against *P*. *falciparum* peptides among the study participants on days 0 (circle) and 42 (square). There was a significant increase in the medians of anti-GLURP (p: <0.0001) and anti-MSP3 (P: <0.0001) IgG levels from day 0 to day 42. However, median anti-HRPII IgG significantly reduced after diagnosis and initiation of treatment.

**Fig 3:**
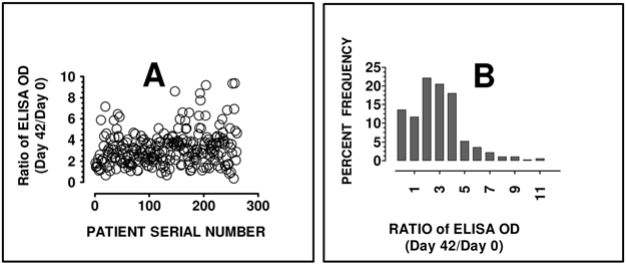
Fold increase in post-infection serum anti-peptide IgG. Sera obtained at diagnosis and 42 days later were tested by ELISA using GLURP, MSP3 and HRPII synthetic peptides as micro titer well-bound antigen coat. For each study participant, the ELISA ODs were added to produce a summative ELISA OD. **A.** The ratio of respective summative ELISA OD for serum collected on day 42 and day 0 (y-axis) was plotted against serial number of study subject (x–axis). ELISA OD represents IgG level while the ratio of the ELISA units for the serum drawn on the 2 days (Day 42/day 0) represents fold-increase in anti-peptide IgG. **B.** Histogram of the ratio (ELISA absorbance for day 42 serum/ ELISA absorbance for day 0 serum). OD: optical density or absorbance.

**Fig 4:**
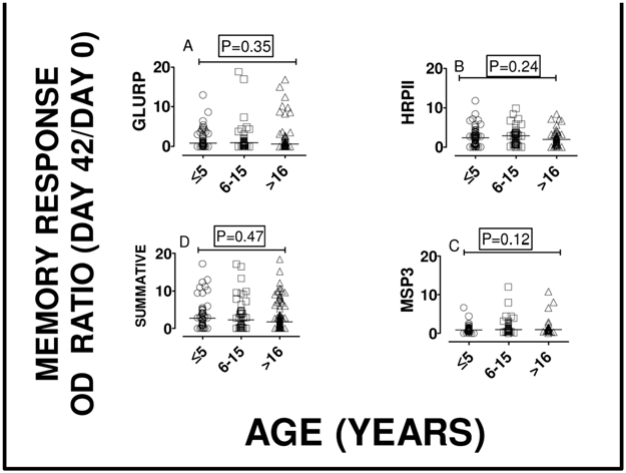
Post-infection immune memory increase of anti-P. falciparum IgG among different age groups. To determine whether post-infection fold-increase of anti-*peptide* IgG varies by age, the medians of summative ELISA ODs (described in Fig 3) for children less than 5 years (< 5), aged from 5 to 16 years and older than 15 years (> 16) were compared. There was no significant difference among the age groups for all three peptides. Kruskal Wallis test results: GLURP (H = 2.1, 2 df, P = 0.35; MSP3 (H = 2.9, 2 df, P = 0.24); HRPII (H = 4.3, 2 df, P = 0.12) and summative ELISA OD (H = 1.5, 2 d.f, P = 0.47).

For many subjects, there was a notable increase in the anti-peptide IgG levels from day 0 to day 42 for all the studied peptides GLURP, MSP3 and HRP II. However, the median anti-peptide IgG for HRP II was significantly reduced from day 0 to day 42. “Fig 2” Anamnestic or memory response was expressed as the ratio of ELISA OD units for IgG levels on day 42 and day 0 or as percent increase. The median anamnestic response as depicted by the above ratio or booster effect was 1.21 fold (interquartile range: 0.96–1.55), namely 20% increase. Based on cumulative ELISA data (where ELISA OD units for all three peptides were added up), about 70% (182/260) of the study participants produced at least 1.2 fold increase in anti-parasite IgG. “Fig 3B” Thirteen study participants (13/260) produced greater than 2-fold post-diagnosis increase in anti-parasite IgG. “Figs 3A and 3B” Only two percent (5/260) of the subjects showed no increase in peptide-specific IgG antibody following a natural *P*. *falciparum* infection. However, for any of the peptides or when the ELISA absorbance units for the three peptides were added together (graph designated ‘Summative’), the post-diagnosis fold-increase in anti-parasite IgG did not significantly vary among the three age groups. “Fig 4”

### Parasite density, CD4^+^ T—cell counts and antibody anamnestic response

**Fig 5:**
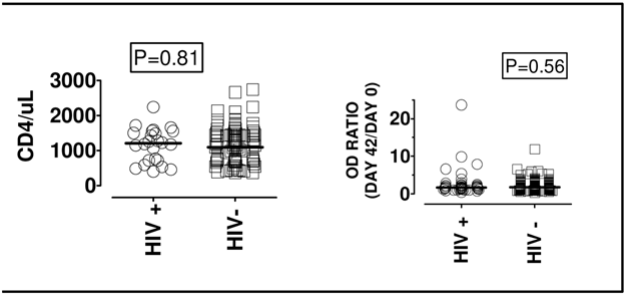
Effect of CD4+ cell count and HIV status on post-infection immune memory increase of anti-P. falciparum IgG. To evaluate the effect of CD4^+^ cell count and HIV status on post-infection increase of *P*. *falciparum* specific antibody, the CD4^+^ cell counts of PLWHAs (n = 24) and HIV negative participants (n = 91) were first determined. There was no significant difference found in CD4^+^ cell counts of HIV negative and PLWHAs (p: 0.81, Mann Whitney test). Medians of the respective ratios for summative ELISA OD (as in Fig 4) of day 42 and day 0 sera from PLWHAs and HIV negative participants were likewise not significantly different (Mann Whitney test, p: 0.56).

The median asexual parasite density for the HIV negative study participants was 6400 PE/μL (IQR: 3000–24000) while the corresponding value for HIV positive subjects was 5600 PE/μL (IQR: 3000–19531), thus indicating insignificant difference in blood *P*. *falciparum* densities between the two groups (p <0.81, Mann-Whitney test). Similarly “Fig 5”, the median CD4^+^ cell count for the HIV ^+^ individuals (n = 24) was 1210/mm^3^ (IQR: 719–1547), while for the HIV negative individuals whose CD4^+^ cells were counted (n = 91) the corresponding value was 1096 /mm^3^ (IQR; 738–1494).

When the post-infection fold increase (absorbance ratio: day 42/day 0) was plotted against the CD4+ cell counts “Fig 5” to determine if there was a relationship between post-infection increase in anti-peptide IgG and CD4^+^ cells counts (results not shown), Spearman’s test revealed no significant relationship between the CD4^+^ cell counts and the memory increase of antibody against peptides MSP-3, GLURP and HRPII. As shown in “Fig 5”, there was no significant difference in the post-infection memory increase of anti-peptide IgG between HIV positive and HIV negative participants (p = 0.56).

## Discussion

### Post-infection antibody immune memory response widely occurs in moderate transmission malaria endemic areas

In work reported here, antibody levels to peptides representing three merozoite antigens (GLURP, MSP3 and HRPII) were assessed among residents of a moderately endemic area in Uganda. Two of these synthetic peptides are conserved epitopes of merozoite surface proteins (GLURP and MSP3) recombinant portions of which comprise GMZ2, a leading blood-stage anti-malarial vaccine candidate that was recently tested at clinical trial phases 1 and 2 in Africa [16, 35]. In the present study, serum antibodies against synthetic peptides of GLURP, MSP3 and HRPII were determined by ELISA tests of blood drawn from patients diagnosed with uncomplicated *P*. *falciparum* acute infection. The mathematical ratio of ELISA OD units for blood collected on the day of malaria diagnosis and 42 days later were considered to represent a rational estimate of the antibody immune memory (anamnestic) response. It was assumed that patients infected with *P*. *falciparum* normally seek treatment at a medical clinic within 2 weeks or so, such that 42 days after diagnosis, the level of blood antibodies on days 0 and 42 would not only provide valid measure of antibody anamnestic response but also enable assessment of post-treatment clinical outcome [29, 30, 36]. The four main findings of this study are 1) the high prevalence of antibodies against conserved epitopes of GLURP, MSP3 and HRPII peptides among PLWHAs and HIV negative participants with uncomplicated malaria in a moderate transmission area of *P*. *falciparum* malaria, 2) the significant increase in GLURP and MSP3 specific antibodies from day 0 and day 42 following *P*. *falciparum* infection and initiation of anti-malarial drug treatment, 3) finding of comparable antibody anamnestic increases among different age groups (below 5 years, 5 to 15 years and above 15) 4) unimpaired antibody-producing anamnestic/memory response among early-stage HIV infected people living with HIV/ AIDS (PLWHA). Whereas there were detectable levels of IgG antibodies recognizing all tested peptides representing *P*. *falciparum* merozoite proteins GLURP, HRP II and MSP3 on day 0 and day 42 among most study subjects, the IgG levels and sero-prevalence for GLURP peptide were higher than the corresponding serum reactivity to MSP3 or to HRPII. This result corroborates earlier studies which have also reported higher IgG levels and sero-prevalence against GLURP in endemic areas [37]. Due to operational limitations, IgG subclass distribution was not investigated in this study although its importance is well recognized [38] and future studies should investigate whether the magnitude and prevalence of post-infection antibody anamnestic responses vary among these immunoglobulin subclasses.

### Inter-individual variability

In the present study, it was found that individuals who showed high anti-GLURP antibody levels did not necessarily have high serum IgG against MSP3 or HRP II, suggesting that predisposing host factors may be the cause of the observed variable sero-reactivity to different antigens. But these results also suggest possible differences in immunogenicity of the peptide epitopes as has been previously observed by others [39]. It is likely that immunoassays using extracts of total parasite lysate may find higher sero-prevalence’s in all residents of comparable endemic study areas since such whole-cell antigen extracts contain thousands of parasite antigens [40–42]. Nevertheless, the finding that all study subjects showed IgG sero-reactivity to at least one of the tested three peptides by day 42 (Fig 1) suggests that antibody priming by blood-stage targeted vaccines such as GMZ2 could be boosted by the few infections experienced annually in moderate-transmission endemic regions. However, the number of previous malaria episodes was not determined as this was a cross-sectional study and this could have given us more information regarding the malaria immune status of the study participants.

Five subjects did not show memory to any of the antigens after clearing an acute malaria episode. It is unlikely that all these individuals have never been exposed to malaria before this episode as they reside in an area of moderate malaria transmission. Several different explanations have been offered by others to explain for an apparent failure to produce antibodies to a defined antigen after a malarial infection. Antigenic polymorphism is common in malarial parasites that could explain low frequencies of responses to certain antigens [43–45]. Similarly, the exposure of an individual to one *P*. *falciparum* “strain” may not be adequate to produce a response on exposure to different epitopes of another “strain” [44]. Others include the poor immunogenicity of a malarial antigen which could lead to a lack of or low and undetectable antibody response. Immune responses to some malarial antigens can be genetically controlled, and this could result in permanently weak responses or non-responsiveness of some individuals.

### Longevity of antibody immune memory

For most participants of this study, antibody anamnestic increase in level of serum anti-parasite IgG from day 0 to day 42 after malaria diagnosis was observed for at least one or two of the three tested peptides. However human B cell memory, mechanisms and durability of antibody immune memory induced either by *P*. *falciparum* natural infections or by vaccine antigens are little understood. There are many reports suggesting that following treatment with antimalarial drugs and resolution of clinical malaria, the levels of antibodies to specific parasite antigens rapidly decline in absence of repeated infections [12, 13]. Indeed, some earlier studies have reported that *P*. *falciparum*-specific Ab immune memory is short-lived, surviving for only few weeks to months [44, 46, 47] compared to anti-viral Ab immune memory which lasts up to tens of years [48]. Nevertheless, more recent studies [49] have now shown that *P*. *falciparum* can also induce and maintain long-lasting parasite specific memory B cells in humans.

### Age dependence of anti-P. falciparum sero-reactivity

In the present study, children aged less than five years had considerably lower levels of specific IgG antibodies against all three peptides studied compared to older children and adults (Fig 1). These results are consistent with those of many other studies and confirm that anti-*P*. *falciparum* antibody immunity increases with age [15, 21, 23].

### Antibody anamnestic response among PLWHAs

While only few (n = 68) people living with human immunodeficiency virus infection/ acquired immuno-deficiency syndrome (HIV/AIDS) (PLWHAs) were enrolled in this study, up to 10% of residents in malaria endemic areas comprise PLWHAs and co infection of *P*. *falciparum* and malaria is important [50, 51]. The duration of how long they were infected is not known. However, as shown (Fig 5), there was no significant difference in the medians of CD4^+^ cell counts for HIV sero-reactive and sero-negative individuals, suggesting that the enrolled PLWHAs were early-diagnosed HIV cases [52, 53] still maintaining high CD4^+^ cell counts. Of these HIV positive individuals, only seven percent (5/68) reported taking Cotrimoxazole prophylaxis therapy and none was on any anti retroviral therapy (ARV). Whereas cell mediated immunity is believed to have a modest effect on the development of naturally acquired resistance to malaria [54–56], some studies have reported loss of memory or activated CD4^+^ T-cells, B cells and plasma cells after primary acute malaria episodes [2, 57, 58].

Accordingly, it was considered useful to compare the antibody post-infection response of early diagnosed PLWHAs and sero-negative subjects in this study. The results shown in Fig 5 are encouraging. Early diagnosed HIV cases, reportedly not yet initiated on HAART regimens, produced as good antibody anamnestic increases as sero-negative individuals. Differences in antibody levels among HIV positives and negative individuals have been reported to occur in later stages of HIV/AIDS when the CD4^+^ cell levels drop to less than 200/uL [59]. Therefore, it would be interesting to investigate antibody anamnestic responses among large numbers of PLWHAs diagnosed with malaria in moderate-transmission malaria areas. Can blood-stage vaccines such as GMZ2 be of value in priming for long-lived boostable anti-malarial immunity among target populations including PLWHAs residing in areas of moderate malaria transmission? The present results (Fig 5) show that HIV sero-negative and early diagnosed PLWHAs with high CD4^+^ T cell counts (study participants in this study all had higher than 350 CD4^+^ cells /uL) possess comparable antibody immune memory towards cognate peptide epitopes of GLURP and MSP3, both constituents of the GMZ2 vaccine candidate [17, 37]. If the present study is carried out among children recently immunized with GMZ2 [16], the results may reveal whether natural infections in moderately low transmission regions can effectively boost the immunity induced by GMZ2 at levels comparable to natural infections[38].

We note that antibody responses to relatively short peptides representing *P*. *falciparum* antigens were measured in this study. However, these synthetic short peptides are unlikely to possess conformation of the native protein and may thus not completely bind antibodies elicited by the native protein. For the same reasons these peptides may also bind non specifically to antibodies and may not exactly reflect the antibody response.

In summary, this study set out to determine the antibody immune memory response elicited by an acute *P*. *falciparum* malaria episode among residents of a moderate-transmission malaria endemic region. The enrolled subjects included predominantly HIV sero-negative individuals and few early-diagnosed HIV sero-positive cases. The results show that 42 days after diagnosis of acute *P*. *falciparum* malaria and initiation of Coartem^TM^ treatment, all the convalescent subjects possess increased serum antibody IgG to at least one of the three tested polypeptides representing important parasite surface proteins GLURP, HRP II and MSP3. A leading blood-stage vaccine candidate, GMZ2 contains GLURP and MSP3 polypeptides. With high blood CD4^+^ cells counts, all PLWHAs enrolled in the study achieved as high antibody anamnestic responses as HIV sero-negative individuals following acute malaria. Detection of increases in *P*. *falciparum* specific IgG antibody in the study subjects after an acute malaria episode suggests that post-infection boosting of specific IgG antibody widely occurs among residents of moderate malaria-transmission endemic areas. This post-infection increase in parasite specific antibody is not influenced by parasite density of the booster infection episode or blood CD4 ^+^cell counts (at least > 350 CD4 /uL) of the convalescent patient.

## References

[1] Lopez AD, Mathers CD, Ezzati M, Jamison DT, Murray CJL. Measuring the Global Burden of Disease and Risk Factors, 1990–2001. 2006. 21250376

[2] Langhorne J, Ndungu FM, Sponaas AM, Marsh K. Immunity to malaria: more questions than answers. Nat Immunol. 2008 7;9(7):725–32. 10.1038/ni.f.205 18563083

[3] Bull PC, Marsh K. The role of antibodies to Plasmodium falciparum-infected-erythrocyte surface antigens in naturally acquired immunity to malaria. Trends Microbiol. 2002 2;10(2):55–8. 11827798 10.1016/s0966-842x(01)02278-8

[4] Osier FH, Fegan G, Polley SD, Murungi L, Verra F, Tetteh KK, et al Breadth and magnitude of antibody responses to multiple Plasmodium falciparum merozoite antigens are associated with protection from clinical malaria. Infection and immunity. 2008 5;76(5):2240–8. 10.1128/IAI.01585-07 18316390 PMC2346713

[5] Garnham PC. Malarial immunity in Africans; effects in infancy and early childhood. Ann Trop Med Parasitol. 1949;43:47–61. 18121270 10.1080/00034983.1949.11685394

[6] Sabchareon A, Burnouf T, Ouattara D, Attanath P, Bouharoun-Tayoun H, Chantavanich P, et al Parasitologic and clinical human response to immunoglobulin administration in falciparum malaria. Am J Trop Med Hyg. 1991 9;45(3):297–308. 1928564 10.4269/ajtmh.1991.45.297

[7] Cohen S, Mc GI, Carrington S. Gamma-globulin and acquired immunity to human malaria. Nature. 1961 11 25;192:733–7. 13880318 10.1038/192733a0

[8] Perraut R, Marrama L, Diouf B, Sokhna C, Tall A, Nabeth P, et al Antibodies to the conserved C-terminal domain of the Plasmodium falciparum merozoite surface protein 1 and to the merozoite extract and their relationship with in vitro inhibitory antibodies and protection against clinical malaria in a Senegalese village. The Journal of infectious diseases. 2005 1 15;191(2):264–71. 15609237 10.1086/426398

[9] Roussilhon C, Oeuvray C, Muller-Graf C, Tall A, Rogier C, Trape JF, et al Long-term clinical protection from falciparum malaria is strongly associated with IgG3 antibodies to merozoite surface protein 3. PLoS medicine. 2007 11 13;4(11):e320 18001147 10.1371/journal.pmed.0040320 PMC2071934

[10] Riley EM, Allen SJ, Wheeler JG, Blackman MJ, Bennett S, Takacs B, et al Naturally acquired cellular and humoral immune responses to the major merozoite surface antigen (PfMSP1) of Plasmodium falciparum are associated with reduced malaria morbidity. Parasite Immunol. 1992 5;14(3):321–37. 1625908 10.1111/j.1365-3024.1992.tb00471.x

[11] al-Yaman F, Genton B, Kramer KJ, Chang SP, Hui GS, Baisor M, et al Assessment of the role of naturally acquired antibody levels to Plasmodium falciparum merozoite surface protein-1 in protecting Papua New Guinean children from malaria morbidity. Am J Trop Med Hyg. 1996 5;54(5):443–8. 8644896 10.4269/ajtmh.1996.54.443

[12] Kinyanjui SM, Bull P, Newbold CI, Marsh K. Kinetics of antibody responses to Plasmodium falciparum-infected erythrocyte variant surface antigens. J Infect Dis. 2003 2 15;187(4):667–74. 12599084 10.1086/373994

[13] Ofori MF, Dodoo D, Staalsoe T, Kurtzhals JA, Koram K, Theander TG, et al Malaria-induced acquisition of antibodies to Plasmodium falciparum variant surface antigens. Infection and immunity. 2002 6;70(6):2982–8. 12010988 10.1128/IAI.70.6.2982-2988.2002 PMC127986

[14] Soe S, Theisen M, Roussilhon C, Aye KS, Druilhe P. Association between protection against clinical malaria and antibodies to merozoite surface antigens in an area of hyperendemicity in Myanmar: complementarity between responses to merozoite surface protein 3 and the 220-kilodalton glutamate-rich protein. Infection and immunity. 2004 1;72(1):247–52. 14688102 10.1128/IAI.72.1.247-252.2004 PMC343946

[15] Akpogheneta OJ, Duah NO, Tetteh KK, Dunyo S, Lanar DE, Pinder M, et al Duration of naturally acquired antibody responses to blood-stage Plasmodium falciparum is age dependent and antigen specific. Infection and immunity. 2008 4;76(4):1748–55. 10.1128/IAI.01333-07 18212081 PMC2292892

[16] Jepsen MP, Jogdand PS, Singh SK, Esen M, Christiansen M, Issifou S, et al The malaria vaccine candidate GMZ2 elicits functional antibodies in individuals from malaria endemic and non-endemic areas. The Journal of infectious diseases. [Clinical Trial, Phase I Randomized Controlled Trial Research Support, Non-U.S. Gov't]. 2013 8 1;208(3):479–88. 10.1093/infdis/jit185 23624363

[17] Mamo H, Esen M, Ajua A, Theisen M, Mordmuller B, Petros B. Humoral immune response to Plasmodium falciparum vaccine candidate GMZ2 and its components in populations naturally exposed to seasonal malaria in Ethiopia. Malaria journal. [Research Support, Non-U.S. Gov't]. 2013;12:51 10.1186/1475-2875-12-51 23383869 PMC3616850

[18] Lousada-Dietrich S, Jogdand PS, Jepsen S, Pinto VV, Ditlev SB, Christiansen M, et al A synthetic TLR4 agonist formulated in an emulsion enhances humoral and Type 1 cellular immune responses against GMZ2—a GLURP-MSP3 fusion protein malaria vaccine candidate. Vaccine. [Research Support, Non-U.S. Gov't]. 2011 4 12;29(17):3284–92. 10.1016/j.vaccine.2011.02.022 21349366

[19] de Souza W. An introduction to the structural organization of parasitic protozoa. Curr Pharm Des. 2008;14(9):822–38. 18473832 10.2174/138161208784041123

[20] Kats LM, Cooke BM, Coppel RL, Black CG. Protein trafficking to apical organelles of malaria parasites—building an invasion machine. Traffic. 2008 2;9(2):176–86. 18047549 10.1111/j.1600-0854.2007.00681.x

[21] Nebie I, Diarra A, Ouedraogo A, Soulama I, Bougouma EC, Tiono AB, et al Humoral responses to Plasmodium falciparum blood-stage antigens and association with incidence of clinical malaria in children living in an area of seasonal malaria transmission in Burkina Faso, West Africa. Infection and immunity. 2008 2;76(2):759–66. 18070896 10.1128/IAI.01147-07 PMC2223475

[22] Zerpa NC, Wide A, Noda J, Bermudez H, Pabon R, Noya OO. Immunogenicity of synthetic peptides derived from Plasmodium falciparum proteins. Experimental parasitology. [Research Support, Non-U.S. Gov't]. 2006 8;113(4):227–34. 16513113 10.1016/j.exppara.2006.01.007

[23] Dodoo D, Theisen M, Kurtzhals JA, Akanmori BD, Koram KA, Jepsen S, et al Naturally acquired antibodies to the glutamate-rich protein are associated with protection against Plasmodium falciparum malaria. The Journal of infectious diseases. 2000 3;181(3):1202–5. 10720556 10.1086/315341

[24] Oeuvray C, Theisen M, Rogier C, Trape JF, Jepsen S, Druilhe P. Cytophilic immunoglobulin responses to Plasmodium falciparum glutamate-rich protein are correlated with protection against clinical malaria in Dielmo, Senegal. Infection and immunity. 2000 5;68(5):2617–20. 10768952 10.1128/iai.68.5.2617-2620.2000 PMC97467

[25] Tall A SC, Perraut R, Fontenille D, Marrama L, Ly AB, Sarr FD, Toure A, Trape JF, Spiegel A, Rogier C, Druilhe P. Assessment of the relative success of sporozoite inoculations in individuals exposed to moderate seasonal transmission. Malar J. 2009;8:161–9. 10.1186/1475-2875-8-161 19604389 PMC2717115

[26] Pinder M MV, Akanmori BD, Genton B, Brown GV. MALVAC 2009: progress and challenges in development of whole organism malaria vaccines for endemic countries, 3–4 June 2009, Dakar, Senegal. Vaccine 2010;28(30):4695–702. 10.1016/j.vaccine.2010.04.091 20470799

[27] World Health Organization. Assessment and monitoring of antimalarial drug efficacy for the treatment of uncomplicated falciparum malaria. Geneva: World Health Organisation 2003.

[28] Prudhomme O'Meara W, Remich S, Ogutu B, Lucas M, Mtalib R, Obare P, et al Systematic comparison of two methods to measure parasite density from malaria blood smears. Parasitology research. [Comparative Study]. 2006 9;99(4):500–4. 16572338 10.1007/s00436-006-0135-x PMC2509584

[29] Ndungu FM, Cadman ET, Coulcher J, Nduati E, Couper E, Macdonald DW, et al Functional memory B cells and long-lived plasma cells are generated after a single Plasmodium chabaudi infection in mice. PLoS Pathog. 2009 12;5(12):e1000690 10.1371/journal.ppat.1000690 20011127 PMC2784955

[30] World Health Organization. WHO guidelines for the treatment of malaria. Geneva World Health Organisation 2006.

[31] Greenwood BM, Armstrong JR. Comparison of two simple methods for determining malaria parasite density. Transactions of the Royal Society of Tropical Medicine and Hygiene. [Comparative Study]. 1991 3-4;85(2):186–8. 1887466 10.1016/0035-9203(91)90015-q

[32] Laman M, Moore BR, Benjamin J, Padapu N, Tarongka N, Siba P, et al Comparison of an assumed versus measured leucocyte count in parasite density calculations in Papua New Guinean children with uncomplicated malaria. Malaria journal. [Research Support, Non-U.S. Gov't]. 2014;13:145 10.1186/1475-2875-13-145 24739250 PMC3991873

[33] Borre MB, Dziegiel M, Hogh B, Petersen E, Rieneck K, Riley E, et al Primary structure and localization of a conserved immunogenic Plasmodium falciparum glutamate rich protein (GLURP) expressed in both the preerythrocytic and erythrocytic stages of the vertebrate life cycle. Mol Biochem Parasitol. 1991 11;49(1):119–31. 1775153 10.1016/0166-6851(91)90135-s

[34] Theisen M, Soe S, Oeuvray C, Thomas AW, Vuust J, Danielsen S, et al The glutamate-rich protein (GLURP) of Plasmodium falciparum is a target for antibody-dependent monocyte-mediated inhibition of parasite growth in vitro. Infect Immun. 1998 1;66(1):11–7. 9423833 10.1128/iai.66.1.11-17.1998 PMC107852

[35] Mordmuller B, Szywon K, Greutelaers B, Esen M, Mewono L, Treut C, et al Safety and immunogenicity of the malaria vaccine candidate GMZ2 in malaria-exposed, adult individuals from Lambarene, Gabon. Vaccine. [Clinical Trial, Phase I Randomized Controlled Trial Research Support, Non-U.S. Gov't]. 2010 9 24;28(41):6698–703. 10.1016/j.vaccine.2010.07.085 20696154 PMC3776947

[36] World Health Organization. Assessment and monitoring of antimalarial drug efficacy for the treatment of uncomplicated falciparum malaria. Geneva: World Health Organisation 2003.

[37] Baumann A, Magris MM, Urbaez ML, Vivas-Martinez S, Duran R, Nieves T, et al Naturally acquired immune responses to malaria vaccine candidate antigens MSP3 and GLURP in Guahibo and Piaroa indigenous communities of the Venezuelan Amazon. Malaria journal. [Research Support, Non-U.S. Gov't]. 2012;11:46 10.1186/1475-2875-11-46 22335967 PMC3296639

[38] Metzger WG, Okenu DM, Cavanagh DR, Robinson JV, Bojang KA, Weiss HA, et al Serum IgG3 to the Plasmodium falciparum merozoite surface protein 2 is strongly associated with a reduced prospective risk of malaria. Parasite immunology. [Research Support, Non-U.S. Gov't]. 2003 6;25(6):307–12. 14507328 10.1046/j.1365-3024.2003.00636.x

[39] Perraut R, Guillotte M, Drame I, Diouf B, Molez JF, Tall A, et al Evaluation of anti-Plasmodium falciparum antibodies in Senegalese adults using different types of crude extracts from various strains of parasite. Microbes Infect. 2002 1;4(1):31–5. 11825772 10.1016/s1286-4579(01)01506-4

[40] Kironde FA, Kumar A, Nayak AR, Kraikov JL. Antibody recognition and isoelectrofocusing of antigens of the malaria parasite Plasmodium yoelii. Infection and immunity. 1991 11;59(11):3909–16. 1937750 10.1128/iai.59.11.3909-3916.1991 PMC258976

[41] Ray P, Sahoo N, Singh B, Kironde FA. Serum antibody immunoglobulin G of mice convalescent from Plasmodium yoelii infection inhibits growth of Plasmodium falciparum in vitro: blood stage antigens of P. falciparum involved in interspecies cross-reactive inhibition of parasite growth. Infection and immunity. 1994 6;62(6):2354–61. 8188358 10.1128/iai.62.6.2354-2361.1994 PMC186518

[42] Avila SL, Tozetto-Mendoza TR, Arruk VG, Ferreira AW. Standardization of procedures of Plasmodium falciparum antigen preparation for serologic tests. Revista do Instituto de Medicina Tropical de Sao Paulo. [Research Support, Non-U.S. Gov't]. 1998 9-10;40(5):309–16. 10030076 10.1590/s0036-46651998000500008

[43] Brown H KD, Barzaga N, Brown GV, Anders RF, Coppel RL. Sequence variation in S-antigen genes of Plasmodium falciparum. Mol Biol Med. 1987;6:365–76. 3325726

[44] Cavanagh DR, Elhassan IM, Roper C, Robinson VJ, Giha H, Holder AA, et al A longitudinal study of type-specific antibody responses to Plasmodium falciparum merozoite surface protein-1 in an area of unstable malaria in Sudan. J Immunol. 1998 7 1;161(1):347–59. 9647243

[45] McBride JS WD, Morgan G. Antigenic diversity in the human malaria parasite Plasmodium falciparum. Science. 1982;217:254–7. 6178159 10.1126/science.6178159

[46] Boutlis CS, Fagan PK, Gowda DC, Lagog M, Mgone CS, Bockarie MJ, et al Immunoglobulin G (IgG) responses to Plasmodium falciparum glycosylphosphatidylinositols are short-lived and predominantly of the IgG3 subclass. J Infect Dis. 2003 3 1;187(5):862–5. 12599061 10.1086/367897

[47] Kinyanjui SM, Conway DJ, Lanar DE, Marsh K. IgG antibody responses to Plasmodium falciparum merozoite antigens in Kenyan children have a short half-life. Malar J. 2007;6:82 17598897 10.1186/1475-2875-6-82 PMC1920526

[48] Amanna IJ, Carlson NE, Slifka MK. Duration of humoral immunity to common viral and vaccine antigens. N Engl J Med. 2007 11 8;357(19):1903–15. 17989383 10.1056/NEJMoa066092

[49] Ndungu FM, Lundblom K, Rono J, Illingworth J, Eriksson S, Farnert A. Long-lived Plasmodium falciparum specific memory B cells in naturally exposed Swedish travelers. European journal of immunology. [Research Support, Non-U.S. Gov't]. 2013 11;43(11):2919–29. 10.1002/eji.201343630 23881859 PMC4114544

[50] Hochman S, Kim K. The Impact of HIV Coinfection on Cerebral Malaria Pathogenesis. Journal of neuroparasitology. 2012;3 22545215 10.4303/jnp/235547 PMC3336366

[51] Tshikuka Mulumba JG, Atua Matindii B, Kilauzi AL, Mengema B, Mafuta J, Eloko Eya Matangelo G, et al Severity of outcomes associated to types of HIV coinfection with TB and malaria in a setting where the three pandemics overlap. Journal of community health. [Research Support, Non-U.S. Gov't]. 2012 12;37(6):1234–8. 10.1007/s10900-012-9559-7 22477668

[52] Wanyenze RK, Kamya MR, Fatch R, Mayanja-Kizza H, Baveewo S, Sawires S, et al Missed opportunities for HIV testing and late-stage diagnosis among HIV-infected patients in Uganda. PloS one. [Research Support, N.I.H., Extramural]. 2011;6(7):e21794 10.1371/journal.pone.0021794 21750732 PMC3130049

[53] Tang H, Mao Y, Shi CX, Han J, Wang L, Xu J, et al Baseline CD4 Cell Counts of Newly Diagnosed HIV Cases in China: 2006–2012. PloS one. 2014;9(6):e96098 10.1371/journal.pone.0096098 24901790 PMC4047021

[54] Marsh K, Otoo L, Hayes RJ, Carson DC, Greenwood BM. Antibodies to blood stage antigens of Plasmodium falciparum in rural Gambians and their relation to protection against infection. Trans R Soc Trop Med Hyg. 1989 5-6;83(3):293–303. 2694458 10.1016/0035-9203(89)90478-1

[55] Butcher GA. HIV and malaria: a lesson in immunology? Parasitol Today. 1992 9;8(9):307–11. 15463649 10.1016/0169-4758(92)90104-a

[56] Whitworth J, Morgan D, Quigley M, Smith A, Mayanja B, Eotu H, et al Effect of HIV-1 and increasing immunosuppression on malaria parasitaemia and clinical episodes in adults in rural Uganda: a cohort study. Lancet. 2000 9 23;356(9235):1051–6. 11009139 10.1016/S0140-6736(00)02727-6

[57] Xu H, Wipasa J, Yan H, Zeng M, Makobongo MO, Finkelman FD, et al The mechanism and significance of deletion of parasite-specific CD4(+) T cells in malaria infection. J Exp Med. 2002 4 1;195(7):881–92. 11927632 10.1084/jem.20011174 PMC2193727

[58] Wykes MN, Zhou YH, Liu XQ, Good MF. Plasmodium yoelii can ablate vaccine-induced long-term protection in mice. J Immunol. 2005 8 15;175(4):2510–6. 16081823 10.4049/jimmunol.175.4.2510

[59] Migot F, Ouedraogo JB, Diallo J, Zampan H, Dubois B, Scott-Finnigan T, et al Selected P. falciparum specific immune responses are maintained in AIDS adults in Burkina Faso. Parasite immunology. [In Vitro Research Support, Non-U.S. Gov't]. 1996 7;18(7):333–9. 9229386 10.1046/j.1365-3024.1996.d01-116.x

